# 1*H*-Pyrrole-2-carboxylic acid

**DOI:** 10.1107/S1600536809014044

**Published:** 2009-04-25

**Authors:** Gui Hong Tang, Dong Dong Li, Gang Huang, Xing Yan Xu, Xiang Chao Zeng

**Affiliations:** a Department of Chemistry, Jinan University, Guangzhou, Guangdong 510632, People’s Republic of China

## Abstract

In the title compound, C_5_H_5_NO_2_, the pyrrole ring and its carboxyl substituent are close to coplanar, with a dihedral angle of 11.7 (3)° between the planes. In the crystal structure, adjacent mol­ecules are linked by pairs of O—H⋯O hydrogen bonds to form inversion dimers. Additional N—H⋯O hydrogen bonds link these dimers into chains extending along the *a* axis.

## Related literature

For pyrroles sourced from marine organisms, see: Faulkner (2002 ▶). For the bioactivity of pyrrole derivatives, see: Banwell *et al.* (2006 ▶); Sosa *et al.* (2002 ▶). For related structures, see: Zeng (2006 ▶); Zeng *et al.* (2007 ▶). For graph-set motifs, see: Bernstein *et al.* (1995 ▶).
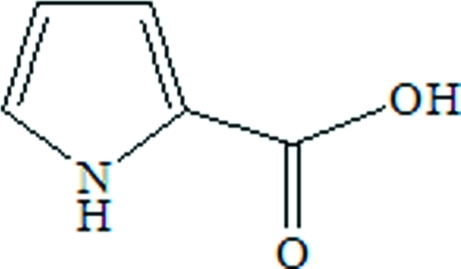


## Experimental

### 

#### Crystal data

C_5_H_5_NO_2_
*M* *_r_* = 111.10
Monoclinic, 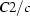

*a* = 14.080 (3) Å
*b* = 5.0364 (10) Å
*c* = 14.613 (3) Å
β = 98.969 (3)°
*V* = 1023.6 (3) Å^3^
*Z* = 8
Mo *K*α radiation
μ = 0.11 mm^−1^
*T* = 173 K
0.42 × 0.40 × 0.37 mm

#### Data collection

Bruker SMART 1K CCD area-detector diffractometer
Absorption correction: multi-scan (*SADABS*; Sheldrick, 1996 ▶) *T*_min_ = 0.954, *T*_max_ = 0.959
2277 measured reflections
1006 independent reflections
875 reflections with *I* > 2σ(*I*)
*R*_int_ = 0.015

#### Refinement

*R*[*F* ^2^ > 2σ(*F* ^2^)] = 0.063
*wR*(*F* ^2^) = 0.191
*S* = 1.06
1006 reflections
74 parameters
H-atom parameters constrained
Δρ_max_ = 0.74 e Å^−3^
Δρ_min_ = −0.73 e Å^−3^

### 

Data collection: *SMART* (Bruker,1999 ▶); cell refinement: *SAINT-Plus* (Bruker, 1999 ▶); data reduction: *SAINT-Plus*; program(s) used to solve structure: *SHELXS97* (Sheldrick, 2008 ▶); program(s) used to refine structure: *SHELXL97* (Sheldrick, 2008 ▶); molecular graphics: *SHELXTL* (Sheldrick, 2008 ▶); software used to prepare material for publication: *SHELXTL*.

## Supplementary Material

Crystal structure: contains datablocks I, global. DOI: 10.1107/S1600536809014044/sj2604sup1.cif

Structure factors: contains datablocks I. DOI: 10.1107/S1600536809014044/sj2604Isup2.hkl

Additional supplementary materials:  crystallographic information; 3D view; checkCIF report

## Figures and Tables

**Table 1:** Hydrogen-bond geometry (Å, °)

*D*—H⋯*A*	*D*—H	H⋯*A*	*D*⋯*A*	*D*—H⋯*A*
N1—H1*A*⋯O1^i^	0.88	2.22	2.951 (3)	141
O2—H2*A*⋯O1^ii^	0.84	2.16	2.986 (3)	166

Symmetry codes: (i) 
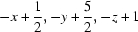
; (ii) 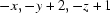.

**Fig. 1.**
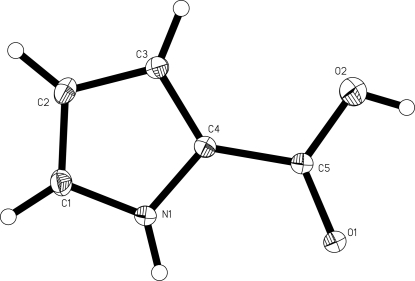
The molecular structure of the title compound, with the atom-numbering scheme. Displacement ellipsoids are drawn at the 30% probability level.

**Fig. 2.**
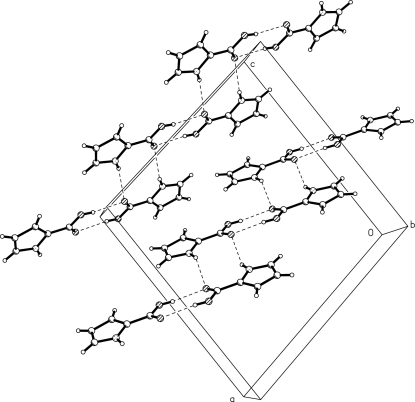
Crystal packing of (I) showing the chains formed by hydrogen bonds (dashed lines).

## supplementary crystallographic information

### Comment

Pyrrole derivatives are well known in many marine organisms (Faulkner, 2002), some show important bioactivities, such as antitumor activity (Banwell *et al.*, 2006) and protein kinase inhibiting activity (Sosa *et al.*, 2002). This is the reason they have attracted our interest. This study is related to our previous structural investigations of methyl 2-(4,5-dibromo-1*H*-pyrrole-2-carboxamido)propionate (Zeng *et al.*, 2007) and 3-bromo-1-methyl-6,7-dihydropyrrolo[2,3-*c*]azepine- 4,8(1*H*,5*H*)-dione (Zeng, 2006). In the crystal structure, molecules of the title compound are linked through N1—H1···O1^i^ hydrogen bonds to form centrosymmetric dimers (Fig. 2) of graph-set motif *R*_2_^2^(10) (Bernstein *et al.*, 1995), which are linked by O2—H2···O1^ii^ hydrogen bonds (another kind of centrosymmetric dimers of graph-set motif *R*_2_^2^(8) are formed), generating chains extending to the *a* axis (also shown in Fig. 2).

### Experimental

The commercially available 1*H*-pyrrole-2-carboxylic acid was dissolved in the mixture of EtOH (80%) and ethyl acetate (20%). Colorless monoclinic crystals suitable for X-ray analysis were obtained when the solution was exposed to the air at room temperature for about 5 d.

### Refinement

All non-H atoms were refined with anisotropic displacement parameters. The H atoms were positioned geometrically [C—H = 0.95Å for CH, O—H = 0.84Å for OH, and N—H = 0.88 Å] and refined using a riding model, with *U*_iso_ = 1.2*U*_eq_ (1.5*U*_eq_ for the methyl group) of the parent atom. In the final difference Fourier map the highest peak (0.74 eÅ^-3^) is 1.01Å from O2 and the deepest hole (-0.73 eÅ^-3^) is 0.61Å from O2.

### Crystal data

**Table:**

C_5_H_5_NO_2_	*F*(000) = 464
*M**_r_* = 111.10	*D*_x_ = 1.442 Mg m^−^^3^
Monoclinic, *C*2/*c*	Melting point: 480 K
Hall symbol: -C 2yc	Mo *K*α radiation, λ = 0.71073 Å
*a* = 14.080 (3) Å	Cell parameters from 1751 reflections
*b* = 5.0364 (10) Å	θ = 2.8–27.0°
*c* = 14.613 (3) Å	µ = 0.11 mm^−^^1^
β = 98.969 (3)°	*T* = 173 K
*V* = 1023.6 (3) Å^3^	Block, colorless
*Z* = 8	0.42 × 0.40 × 0.37 mm

### Data collection

**Table:**

Bruker SMART 1K CCD area-detector diffractometer	1006 independent reflections
Radiation source: fine-focus sealed tube	875 reflections with *I* > 2σ(*I*)
graphite	*R*_int_ = 0.015
φ and ω scans	θ_max_ = 26.0°, θ_min_ = 2.8°
Absorption correction: multi-scan (*SADABS*; Sheldrick, 1996)	*h* = −17→13
*T*_min_ = 0.954, *T*_max_ = 0.959	*k* = −6→6
2277 measured reflections	*l* = −14→18

### Refinement

**Table:**

Refinement on *F*^2^	Primary atom site location: structure-invariant direct methods
Least-squares matrix: full	Secondary atom site location: difference Fourier map
*R*[*F*^2^ > 2σ(*F*^2^)] = 0.063	Hydrogen site location: inferred from neighbouring sites
*wR*(*F*^2^) = 0.191	H-atom parameters constrained
*S* = 1.06	*w* = 1/[σ^2^(*F*_o_^2^) + (0.1108*P*)^2^ + 3.3345*P*] where *P* = (*F*_o_^2^ + 2*F*_c_^2^)/3
1006 reflections	(Δ/σ)_max_ = 0.001
74 parameters	Δρ_max_ = 0.74 e Å^−^^3^
0 restraints	Δρ_min_ = −0.73 e Å^−^^3^

### Special details

**Table:**

Geometry. All e.s.d.'s (except the e.s.d. in the dihedral angle between two l.s. planes) are estimated using the full covariance matrix. The cell e.s.d.'s are taken into account individually in the estimation of e.s.d.'s in distances, angles and torsion angles; correlations between e.s.d.'s in cell parameters are only used when they are defined by crystal symmetry. An approximate (isotropic) treatment of cell e.s.d.'s is used for estimating e.s.d.'s involving l.s. planes.
Refinement. Refinement of *F*^2^ against ALL reflections. The weighted *R*-factor *wR* and goodness of fit *S* are based on *F*^2^, conventional *R*-factors *R* are based on *F*, with *F* set to zero for negative *F*^2^. The threshold expression of *F*^2^ > σ(*F*^2^) is used only for calculating *R*-factors(gt) *etc*. and is not relevant to the choice of reflections for refinement. *R*-factors based on *F*^2^ are statistically about twice as large as those based on *F*, and *R*- factors based on ALL data will be even larger.

### Fractional atomic coordinates and isotropic or equivalent isotropic displacement parameters (Å^2^)

**Table:**

	*x*	*y*	*z*	*U*_iso_*/*U*_eq_	
O1	0.12435 (12)	1.1503 (3)	0.53422 (12)	0.0223 (5)	
C4	0.23786 (16)	0.8483 (5)	0.61313 (15)	0.0176 (6)	
O2	0.07382 (14)	0.7350 (4)	0.56343 (15)	0.0373 (6)	
H2A	0.0220	0.7923	0.5336	0.056*	
N1	0.31542 (14)	1.0100 (4)	0.61094 (15)	0.0216 (6)	
H1A	0.3144	1.1614	0.5808	0.026*	
C3	0.26837 (17)	0.6325 (5)	0.66849 (17)	0.0208 (6)	
H3	0.2299	0.4879	0.6828	0.025*	
C5	0.14189 (16)	0.9228 (5)	0.56657 (15)	0.0173 (6)	
C2	0.36767 (18)	0.6681 (5)	0.69974 (17)	0.0245 (6)	
H2	0.4085	0.5521	0.7393	0.029*	
C1	0.39405 (17)	0.9010 (6)	0.66242 (18)	0.0251 (6)	
H1	0.4570	0.9740	0.6712	0.030*	

### Atomic displacement parameters (Å^2^)

**Table:**

	*U*^11^	*U*^22^	*U*^33^	*U*^12^	*U*^13^	*U*^23^
O1	0.0198 (9)	0.0190 (10)	0.0273 (10)	−0.0004 (7)	0.0008 (7)	0.0048 (7)
C4	0.0184 (12)	0.0182 (12)	0.0167 (11)	−0.0004 (9)	0.0039 (9)	−0.0005 (9)
O2	0.0298 (11)	0.0331 (12)	0.0472 (13)	−0.0029 (9)	0.0002 (10)	0.0044 (10)
N1	0.0191 (10)	0.0196 (11)	0.0253 (11)	−0.0027 (8)	0.0013 (8)	0.0062 (8)
C3	0.0210 (12)	0.0198 (12)	0.0216 (12)	0.0003 (9)	0.0035 (9)	0.0020 (9)
C5	0.0192 (12)	0.0164 (11)	0.0167 (11)	−0.0002 (9)	0.0042 (9)	−0.0008 (9)
C2	0.0220 (13)	0.0291 (14)	0.0215 (12)	0.0052 (10)	0.0009 (9)	0.0038 (10)
C1	0.0174 (12)	0.0318 (14)	0.0256 (13)	−0.0013 (10)	0.0019 (9)	0.0030 (11)

### Geometric parameters (Å, °)

**Table:**

O1—C5	1.250 (3)	N1—H1A	0.8800
C4—N1	1.367 (3)	C3—C2	1.413 (3)
C4—C3	1.383 (3)	C3—H3	0.9500
C4—C5	1.464 (3)	C2—C1	1.369 (4)
O2—C5	1.342 (3)	C2—H2	0.9500
O2—H2A	0.8400	C1—H1	0.9500
N1—C1	1.354 (3)		
			
N1—C4—C3	107.8 (2)	O1—C5—O2	122.4 (2)
N1—C4—C5	121.3 (2)	O1—C5—C4	121.6 (2)
C3—C4—C5	130.8 (2)	O2—C5—C4	116.0 (2)
C5—O2—H2A	109.5	C1—C2—C3	107.2 (2)
C1—N1—C4	109.4 (2)	C1—C2—H2	126.4
C1—N1—H1A	125.3	C3—C2—H2	126.4
C4—N1—H1A	125.3	N1—C1—C2	108.6 (2)
C4—C3—C2	106.9 (2)	N1—C1—H1	125.7
C4—C3—H3	126.5	C2—C1—H1	125.7
C2—C3—H3	126.5		
			
C3—C4—N1—C1	0.7 (3)	N1—C4—C5—O2	171.9 (2)
C5—C4—N1—C1	177.3 (2)	C3—C4—C5—O2	−12.3 (4)
N1—C4—C3—C2	−0.2 (3)	C4—C3—C2—C1	−0.3 (3)
C5—C4—C3—C2	−176.4 (2)	C4—N1—C1—C2	−0.9 (3)
N1—C4—C5—O1	−10.0 (3)	C3—C2—C1—N1	0.7 (3)
C3—C4—C5—O1	165.7 (2)		

### Hydrogen-bond geometry (Å, °)

**Table:**

*D*—H···*A*	*D*—H	H···*A*	*D*···*A*	*D*—H···*A*
N1—H1A···O1^i^	0.88	2.22	2.951 (3)	141
O2—H2A···O1^ii^	0.84	2.16	2.986 (3)	166

Symmetry codes: (i) −*x*+1/2, −*y*+5/2, −*z*+1; (ii) −*x*, −*y*+2, −*z*+1.
